# Cervical Cancer Screening Program by Visual Inspection: Acceptability and Feasibility in Health Insurance Companies

**DOI:** 10.1155/2015/798453

**Published:** 2015-06-18

**Authors:** Apollinaire G. Horo, Judith Didi-Kouko Coulibaly, Abdoul Koffi, Boris Tchounga, Konan Seni, Kacou Edèle Aka, Mamourou Kone

**Affiliations:** ^1^Department of Gynaecology and Obstetrics, Teaching Hospital of Yopougon, Félix Houphouët Boigny (FHB) University, Abidjan, Côte d'Ivoire; ^2^Oncology Department, Félix Houphouët Boigny (FHB) University, Teaching Hospital of Treichville, Abidjan, Côte d'Ivoire; ^3^PACCI Program, IeDEA West Africa Collaboration, Abidjan, Côte d'Ivoire

## Abstract

*Objective*. To assess willingness to participate and diagnostic accuracy of visual inspection for early detection of cervical neoplasia among women in a health insurance company.* Patients and Method*. Cervical cancer screening was systematically proposed to 800 women after consecutive information and awareness sessions. The screening method was visual inspection with acetic acid (VIA) or Lugol's iodine (VILI).* Results*. Among the 800 identified women, 640 (82%) have accepted the screening, their mean age was 39 years, and 12.0% of them were involved in a polygamist couple. 28.2% of women had prior cervical screening. VIA has been detected positive in 5.9% of women versus 8.6% for VILI. The sensitivity was 72.9% and specificity was 95.2% for VIA versus 71.2% and 97.3% for VILI respectively. The histological examination highlighted a nonspecific chronic cervicitis in 4.6%, CIN1 lesions in 5.91%, and CIN2/3 in 1.2% of the cases.* Conclusion*. Cervical cancer screening by visual inspection showed appropriate diagnostic accuracy when used to detect early cervical lesions. It is a simple and easy to perform method that could be introduced progressively in the health insurance policy while waiting for a national screening program.

## 1. Introduction

Each year, about 490 000 new cases of cervical cancer occurred worldwide, mainly in developing countries (80%) [1]. In these countries, cervical cancer is the second most common cancer among women after breast cancer and the leading cause of cancer deaths [1, 2]. In developed countries, the incidence and mortality of cervical cancer decreased significantly, due to implementation of organized cervical cancer screening programs [2]. Such programs are not yet implemented in many resources constrained settings, where access to early detection of cervical neoplasia is still challenging and most patients are diagnosed at an advanced stage [3]. In Côte d'Ivoire, cervical cancer screening is based only on cytology and is not organized in a subsidized program; the cost is then supported by women themselves. Since 2005 the World Health Organization (WHO) recommended visual inspection of cervix with acetic acid (VIA) or Lugol's iodine (VILI) as alternative screening techniques for the detection of cervical precancerous lesions. Many field evaluation surveys reported that visual inspection demonstrated diagnostic accuracy close to cytology and was more affordable and easy to perform for nonmedical health workers in developing countries [2, 4]. Côte d'Ivoire is now planning to implement a national cervical cancer screening program and the screening technic as well as the entry point to screen a maximum of women that are questions of interest.

In order to propose solutions to national cancer control program, we assess the willingness to participate in a screening program among women working in a health insurance company and the diagnostic accuracy of visual inspection as screening method in Côte d'Ivoire (West Africa).

## 2. Method

### 2.1. Study Design

A cross-sectional survey was conducted among women working in health insurance companies from January to December 2010.

### 2.2. Study Population

During this period, cervical screening by VIA and VILI was proposed to all the 800 identified women working in health insurance companies in Abidjan.

### 2.3. Visual Inspection with Acetic Acid (VIA)

VIA was done with 5% freshly prepared dilute acetic acid. The color changes were observed one minute after the application of the acetic acid. The results of the VIA were recorded as suspect cancer, positive (for acetic white lesion at the squamocolumnar junction), negative (no acetowhite lesion), or squamocolumnar junction not visible.

### 2.4. Visual Inspection with Lugol's Iodine

Following VIA, visual inspection with Lugol's iodine (VILI) was also performed. A positive VILI test was characterized by a dense, thick, bright, mustard-yellow, or saffron-yellow iodine nonuptake area seen in the transformation zone.

Results of VIA and VILI were both classified as negative, positive, or indicative only of ICC according to the WHO Guidelines for Screening and Treatment of Precancerous Lesions [5].

### 2.5. Colposcopy

Patients who were positive at either of the screening tests underwent a colposcopy exam. Cervical biopsy was performed to positives women. Histopathological findings were categorized into five classes: normal or nonneoplastic changes, cervical intraepithelial neoplasia grade 1 (CIN1) including HPV changes, CIN2, CIN3, and ICC (invasive cervical cancer).

### 2.6. Data Collection and Analysis

A structured survey form was used to collect data on the willingness to participate and the results of screening when it was performed. The analysis was performed using Epi info software, and qualitative variables were presented in rough values and in percentage while quantitative variables were presented in means and standard deviation.

### 2.7. Patient's Outcomes

When visual inspection was negative, the patient was reassured and called for a new test within a period of 3 years. For CIN2/3 lesions (cervical intraepithelial neoplasia grade 2/3) a cryotherapy or a loop electrosurgical excision (LEE) procedure was performed the same day or delayed for 24 hours. CIN1 benefited from annual monitoring, and ICCs were treated by Wertheim's hysterectomy when the patient agreed.

### 2.8. Ethical Statements

This study was approved by the Ethics Committee of the University Hospital of Yopougon, and all the participants gave a written consent before being included in the study.

## 3. Results

### 3.1. Demographics Characteristics

Among the 800 women identified in health insurance companies, 640 (80%) accepted to participate in the study and to have a cervical cancer screening by visual inspection.

The median age of the participant was 39 [21–59] years, 603 (94.2%) were married, and 12.0% were in a polygamist couple. The average number of childbirth per woman was 2.2 and 28.2% of women had already a cervical cancer screening (Table 1).

### 3.2. Results of Cervical Cancer Screening

Among the 640 women tested, VIA remains positive in 5.9% of cases while VILI was positive in 8.6% and colposcopy was abnormal in 9.3%. VIA showed 72.9% sensitivity and 95.2% specificity, while VILI showed 71.2% and 97.3%, respectively (Table 2). Histological examination of biopsy showed nonspecific chronic cervicitis in 4.6% (26 cases), CIN1 (5.9%), and CIN2/3 lesions: 1.2% (Table 3).

## 4. Discussion

This study reports the results of a cervical cancer screening among 640 women working in health insurance companies. Globally the willingness to participate was 80%, and less than 10% of the tests performed remain abnormal, principally due to nonspecific chronic cervicitis and CIN1.

Most of the participants were aged 30 to 49 years and the youngest was 21 years old. In Côte d'Ivoire, like in many other African countries there is no recommendation concerning the beginning age for cervical cancer screening. In France, the High Authority for Health (HAS) recommends starting pap smear at 25 years because of the high proportion of transient HPV infections, while the American Gynecologist Association leans toward a first smear at 21 years (or 3 years after first sexual intercourse) based therefore on a period of 36 months to see lesions CIN3 [6, 7]. All gynecologist societies agree that performing cervical cancer screening on teenager age 21 leads to anxiety, increased costs, and inappropriate treatments [8, 9].

Furthermore, we investigated the determinants of cervical cancer in our population. Regarding obstetric history, participants were pauciparous in 22.9% of cases like Blumenthal found in Ghana [1]. However in Cameroon in a rural area, Tebeu et al. [10] reported an average rate of 5.6 children during a one-time screening campaign. It is established that the multiparous and the precocity of sexual intercourse are risk factors for cervical cancer [11]. But in our study, there were mainly young, active, and literate pauciparous women who participated in the screening program. Moreover, 7.5% of women were involved in a polygamist couple, and this factor is often underestimated because there is a form of polygamy not formalized in most African countries. In Thailand, Cameroon, and Ghana, the partner was widely polygamist [1, 6, 10] with at least two sexual partners.

In our survey only 21.4% of women had performed a cervical cancer screening prior to the study with a delay of several years. This is related to the absence of national policies for early detection of cervical neoplasia. So far only a few hospitals offer cervical cancer screening by visual inspection. However an extensive provincial health workers training program is underway for cervical screening and treatment of cervical lesions.

### 4.1. Coverage Rates

Women's participation was 80%, and it is the minimum threshold for cervical cancer screening to impact on cervical cancer mortality. This result seems to be unsatisfactory considering the awareness campaign, before the study, and can predict the low interest in the general population.

### 4.2. Performance of Visual Inspection

Our study showed a positive VIA in 5.8% and 8.6% for the VILI. In Angola, Muwonge [9] did the same study on a population of 8851 women. The VIA and VILI were, respectively, positive in 6.6% and 5.2% of the cases. In Ghana, the test was positive in 13.2% of women [1]. The figures of precancerous lesions vary across the authors around 10%.

The sensitivity of the VIA was 72.9% and its specificity was 95.2% in our survey. These values are close to those found by many authors [1, 4, 12–14]. Acetic acid is cheaper and available while Lugol's iodine is more expensive. Because of the limited resources, some countries such as Ghana, Angola, China, and India fail to realize combined screening with acetic acid and Lugol's iodine. Thus they showed that VIA alone is sufficient for the detection of cervical precancerous lesions. Its sensitivity and specificity vary, respectively, between 66 and 96% and 64 and 98% according to Basu et al. and JHPIEGO (Johns Hopkins Program for International Education in Gynecology and Obstetrics) [15]. Several authors have conducted surveys in low resource countries, performing visual inspection with acetic acid compared to cytology.

Arbyn et al. [13] realized multicentric studies in a low setting population in 2008 using only acetic acid for cervical screening. They noted 79% for sensibility and 85% for specificity.

Fifteen studies in total were reviewed by Gafirikin et al. [14]; they reported sensitivity ranged between 66% and 96% and specificity between 64% and 98%. Finally, they suggested that VIA has the potential to be a cervical cancer screening tool, especially in low resource settings. Besides, Basu et al. [15] presented a comparison between VIA and cytology. The sensitivity of VIA to detect CIN 2-3 lesions was 55.7%, and the specificity was 82.1%. The sensitivity and specificity of cytology were 29.5% and 92.3%, respectively. Ardahan and Temel [16] in Turkey reported 85.29% sensitivity and 68.75% specificity when comparing colposcopy and VIA.

Furthermore we observed for VILI 71.2% sensitivity and a specificity of 97.3%. These results are consistent with others found in the literature, showing the superiority of Lugol's compared to acetic acid [2, 7]. However, using Lugol's is still challenging due to its cost and accessibility. In some remote parts of India repeated shortages have imposed the single use of acetic acid [4].

Regarding treatment, according to WHO recommendations cryotherapy should be used for CIN1 and conization or LEE for CIN2/3 [4, 16]. Treatment of CIN2 by LEE is still discussed due to obstetric complications when these lesions are found in young women between 25 and 35 years [6, 7, 17]. The authors argue that the risk of cancer is very low in this age group where CIN is frequent. Monitoring is recommended, but the treatment is acceptable. We systematically treated lesions due to strong possibility of being lost to follow-up (36% in a similar study in an HIV clinic) [18].

## 5. Conclusion

Cervical cancer screening by visual inspection showed appropriate diagnostic accuracy when used to detect early cervical lesions. It is a simple and easy to perform method that could be introduced progressively in the health insurance policy while waiting for a national screening program.

## Figures and Tables

**Table 1:** Demographic characteristics of participants of the screening.

	Frequency (*n*)	Percentage (%)
Age (years)		
20–29	69	6.6
30–39	266	25.8
40–49	216	20.9
≥50	90	8.7
Parity		
0–2 (pauciparous)	236	22.9
3-4 (multiparous)	331	51.7
5 (high multiparous)	73	7.1
Polygamy		
Yes	77	12.0
No	401	62.7
Does not know	125	19.5
Separate	37	5.8
Delay screening		
0–3 years	66	10.3
3 years	71	11.1
Never detected	503	78.6

**Table 2:** Diagnostic performances of visual inspection.

	Sensitivity (%)	Specificity (%)
VIA (visual inspection with acetic acid)	72.9	95.2
VILI (visual inspection with Lugol's iodine)	71.2	97.3

**Table 3:** Histological results.

Lesions	Frequency (*n*)	Percentage (%)
Nonspecific chronic cervicitis	26	4.6
CIN1	33	5.9
CIN2/3	7	1.2

Total	66	11.7
